# The Use of DArTseq Technology to Identify Markers Linked to Genes Responsible for Seed Germination and Seed Vigor in Maize

**DOI:** 10.3390/ijms232314865

**Published:** 2022-11-28

**Authors:** Bartosz Nowak, Agnieszka Tomkowiak, Jan Bocianowski, Aleksandra Sobiech, Roksana Bobrowska, Przemysław Łukasz Kowalczewski, Marianna Bocianowska

**Affiliations:** 1 Smolice Plant Breeding Sp. z o. o. IHAR Group, Smolice 146, 63-740 Kobylin, Poland; 2 Department of Genetics and Plant Breeding, Poznań University of Life Sciences, Dojazd 11, 60-632 Poznań, Poland; 3 Department of Mathematical and Statistical Methods, Poznań University of Life Sciences, Wojska Polskiego 28, 60-637 Poznań, Poland; 4 Department of Food Technology of Plant Origin, Poznań University of Life Sciences, 31 Wojska Polskiego St., 60-624 Poznań, Poland; 5 Faculty of Chemical Technology, Poznań University of Technology, Piotrowo 3A, 60-965 Poznań, Poland

**Keywords:** inbred lines, maize, seed germination, seed vigor, molecular markers, next-generation sequencing

## Abstract

Seed vigor and seed germination are very important traits, determined by several factors including genetic and physical purity, mechanical damage, and physiological condition, characterized by maintaining a high seed vigor and stable content after storage. The search for molecular markers related to improvement in seed vigor under adverse condition is an important issue in maize breeding currently. Higher sowing quality of seeds is necessary for the development of the agriculture production and better ability to resist all kinds of adversity in the seeds’ storage. Condition is a very important factor affecting the yield of plants, thanks to the construction of their vitality. Identification of molecular markers associated with seed germination and seed vigor may prove to be very important in the selection of high-yielding maize varieties. The aim of this study was to identify and select new markers for maize (SNP and SilicoDArT) linked to genes influencing the seed germination and seed vigor in inbred lines of maize (*Zea mays* L.). The plant material used for the research was 152 inbred maize lines. The seed germination and seed vigor were analyzed. For identification of SNP and SilicoDArT markers related to the seed germination and seed vigor, the SilicoDarT technique developed by Diversity Arrays Technology was used. The analysis of variance indicated a statistically significant differentiation between genotypes for both observed traits. Positive (*r* = 0.41) correlation (*p* < 0.001) between seed germination and seed vigor was observed. As a result of next-generation sequencing, the molecular markers SilicoDArT (53,031) and SNP (28,571) were obtained. Out of 81,602 identified SilicoDArT and SNP markers, 15,409 (1559 SilicoDArT and 13,850 SNP) were selected as a result of association mapping, which showed them to be significantly related to the analyzed traits. The 890 molecular markers were associated with seed vigor, and 1323 with seed germination. Fifty-six markers (47 SilicoDArT and nine SNP) were significant for both traits. Of these 56 markers, the 20 most significant were selected (five of these markers were significant at the level of 0.001 for seed vigor and at the level of 0.05 for seed germination, another five markers were significant at the level of 0.001 for seed germination and at the level of 0.05 for seed vigor, five markers significant at the level of 0.001 only for seed vigor and five significant at the level of 0.001 only for seed germination also selected). These markers were used for physical mapping to determine their location on the genetic map. Finally, it was found that six of these markers (five silicoDArT—2,435,784, 4,772,587, 4,776,334, 2,507,310, 25,981,291, and one SNP—2,386,217) are located inside genes, the action of which may affect both seed germination and seed vigor. These markers can be used to select genotypes with high vigor and good seed germination.

## 1. Introduction

Caring for the seed vigor and germination of cereals, including corn, very often escapes the attention of farmers who focus primarily on fighting weeds, fungal diseases, and pests [1]. It is well known, however, that the condition of plants is a very important factor influencing the number of yields, and herbicides and fungicides are not sufficient in building plant vitality [2,3]. The seed vigor of plants and the ability to have undisturbed development under stressful conditions is possible to achieve, inter alia, thanks to natural predispositions, which are genetically determined [4,5]. Currently, many companies offer preparations in the form of a seed dressing, which not only improve the overall health of plants but also affect their vigor. Unfortunately, from the ecological point of view, the currently used high-toxic synthetic compounds may remain in the soil for a long time in some cases. Therefore, the natural vitality of plants is a very desirable trait [6,7,8,9].

A grain seed with high vigor is characterized by fast and uniform emergence, while poor vigor results in slow and staggered emergence. These traits affect the speed of plant aging and resistance to adverse weather conditions, as well as yield. Improving the quality parameters of crop plants through attention to vigor and stress resistance is possible through proper genotype selection in the process of breeding new varieties, which increases the chances of success in the form of high-quality yields [2].

In the era of rapid development of molecular biology tools, it is important to identify markers related to genes responsible for seed vigor and seed germination. These activities are aimed at facilitating the selection of genotypes characterized by high seed vigor and good seed germination [10].

Rapid advances in Next Generation Sequencing (NGS) have made it possible to sequence the genome of many crops. High-throughput genotyping methods such as GBS and SNP enable rapid genome profiling to provide growers with detailed information on traits relevant to cultivation. High-resolution genotyping may therefore be the key to revitalizing phenotypic diversity in response to climate change [11].

The identification of genomic regions associated with different phenotypic traits, including vigor and germination, is fundamental to understanding and manipulating these traits. The advent of high-throughput genome sequencing technologies and computational approaches have led to the sequencing of reference genomes of various crops. Genome-wide association studies (GWAS) have therefore become a powerful methodology for studying genetic variation and identifying the relationship between a trait and the underlying genetic variation by exploiting historical recombination events [12].

Sanghamitra et al. [13] described the relationship of molecular markers with eleven physiological traits that affect seed vigor. Genotypes present within groups and sub-groups showed similarity for their physiological traits. Strong association of the markers with physiological parameters namely RM167 for seed vigor index-I; RM7364 and RM235 for seed vigor index-II; RM440, RM223 for seedling dry weight; RM 256, RM25181, RM6547, RM328, RM201 for rate of root growth; RM 20, RM13335, RM216 for rate of shoot growth while RM20A and RM201 for absolute growth rate were detected by both GLM and MLM analyses. Markers detected in this association analysis may be use for future seed vigor improvement in rice.

Sahoo et al. [14] they analyzed eighteen physio-biochemical traits influencing seed vigor for their association with molecular markers using a mini core set constituted from 120 germplasm lines. Strong positive correlation of seed vigor was observed with amylose content, total anthocyanin content, catalase, total phenolic content, and total flavonoid content while a negative association was observed for gamma-oryzanol content. High gene diversity (0.7169) and informative markers value (0.6789) were estimated from the investigation.

Mahender et al. [15] used molecular markers to select genotypes characterized by high seed vigor. A marker-assisted selection approach facilitated the efficient and precise transfer of genes/QTL(s) to many crop species.

We know that prolonged storage generally reduces seed viability and vigor, although the rate of deterioration varies among species and environmental conditions. Garza-Caligaris et al. [16] suggest a possible ageing molecular marker: At3g08030 mRNA. At3g08030 is a member of the DUF642 highly conserved family of cell-wall-associated proteins that is specific for spermatophytes [16].

Mesocotyl length (MES) is an important feature influencing the emergence of maize seedlings after deep sowing and is closely related to abiotic stress. Understanding the constitutive QTLs (cQTLs) and candidate genes for MES is therefore of great importance in marker-assisted breeding (MAS). In the course of their research, Froidure et al. [17] found that MYB transcription factors may play an important role in regulating germination from deep soil layers. Research on plant MYB transcription factors involved in plant growth and development has focused mainly on *Arabopsis thaliana*. The discovery of key germination genes in deep sowing is of great importance for the cultivation of high-germination maize varieties in the soil [18].

Genetic analysis of traits influencing the vigor and germination of seeds is important not only in terms of the end result in the form of a high yield, but also in the context of biodiversity protection. For a long time, we have been observing biodiversity crisis. The value of ex situ conservation has been increasingly acknowledged in international treaties and legislations. Seed banks are a good way of conserving biodiversity, providing that seeds are of high quality and at maximum viability. However, despite the number of established ex situ facilities, there is little information on seed viability in botanic garden seed banks.

The condition of plants is an important factor influencing the yield and vitality of plants. Identification of molecular markers linked with the seed germination and seed vigor will allow for a quick selection of varieties and lines with increased yield potential. The use of molecular markers will also allow for quick selection of genotypes with longer seed viability, which can be successfully stored in Gene Banks.

The aim of this study was to identify new markers (SNP and SilicoDArT) uses for maize selection linked to genes influencing the seed germination and seed vigor in maize.

## 2. Results

### 2.1. Analysis of the Seed Germination and Seed Vigor of Inbred Maize Lines

Both the observed traits (seed germination and seed vigor) had normal distribution. Analysis of variance indicated that the main effects of lines was significant for both traits of study. Mean values of seed germination varied from 3 (for line no. 137) to 9 (for 60 lines) with an average of 8.12. Mean values of seed vigor varied from 3 (for lines: no. 50 and no. 85) to 9 (for 57 lines) with an average of 7.55. Positive (*r* = 0.41), statistically significant (*p* < 0.001) correlation between seed germination and seed vigor was observed.

### 2.2. Association Mapping using GWAS Analysis

A total of 152 lines were sent for next-generation sequencing and were also analyzed in the field. As a result of next-generation sequencing, a total of 81,602 molecular markers (53,031 SilicoDArT and 28,571 SNP) were obtained, of which 15,409 (1559 SilicoDArT and 13,850 SNP) were selected as a result of association mapping, which showed them to be significantly related to the analyzed traits.

**Figure 1:**
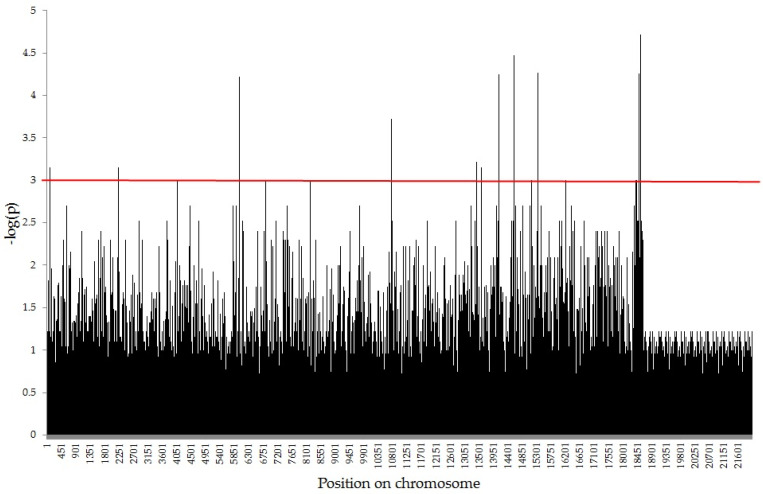
Manhattan plots for seed vigor.

The 890 molecular markers (758 SilicoDArT and 132 SNP) were associated with seed vigor (Figure 1 and Table 1). Effects of particular markers varied from −0.801 (for SilicoDArT marker 25,947,631) to 0.714 (for SNP marker 4,770,911), with an average of −0.004 (0.0015 for SilicoDArT and −0.036 for SNP). Percentage variance accounted by particular markers varied from 2% (for 89 markers) to 7.6% (for SilicoDArT marker 4,580,898), with an average of 3.11% (3.01% for SilicoDArT and 3.06% for SNP).

**Figure 2:**
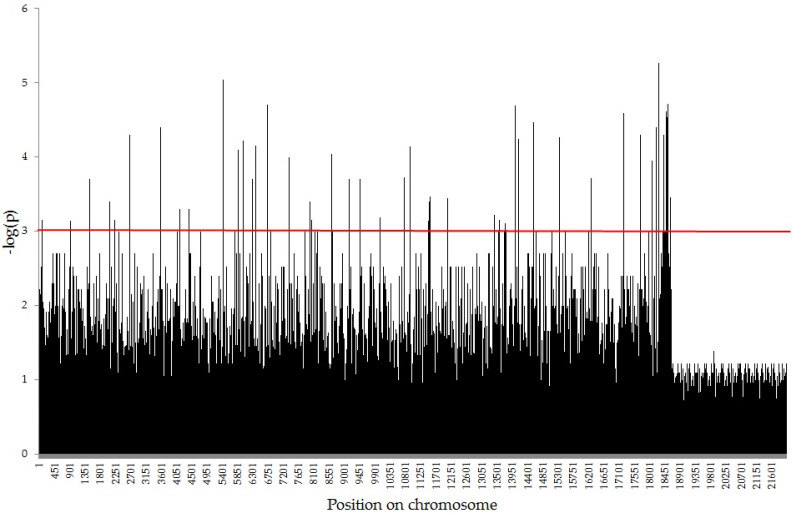
Manhattan plots for seed germination.

The 1323 (1115 SilicoDArT and 208 SNP) with seed germination (Figure 2). Effects of particular markers varied from −0.639 (for SilicoDArT marker 2,382,757) to 0.637 (for SNP marker 2,386,217), with an average of −0.081 (−0.088 for SilicoDArT and −0.046 for SNP). Percentage variance of seed germination accounted by particular markers varied from 1.9% (for 56 markers) to 10.2% (for SilicoDArT marker 2,382,757), with an average of 3.16% (3.14% for SilicoDArT and 3.3% for SNP).

Fifty-six markers (47 SilicoDArT and nine SNP) were significant for both traits (Table 2). Effects of particular markers varied from −0.737 (for SilicoDArT marker 4,589,607 for seed vigor) and −0.540 (for SilicoDArT marker 4,765,935 for seed germination) to 0.748 (for SilicoDArT marker 7,059,320 for seed vigor) and 0.455 (for SilicoDArT marker 4,772,587 for seed germination), with an average of −0.065 and −0.075 for seed vigor and seed germination, respectively. Percentage variance accounted by particular markers varied from 1.9% (for seed germination) and 2.0% (for seed vigor) to 6.6% (for seed vigor) and 6.7% (for seed germination), with an average of 3.23% and 2.64% for seed vigor and seed germination, respectively (Table 2).

Selected markers, significant at 0.001 level with seed vigor, are presented in Table 3. Effects of particular markers varied from −0.801 (for SilicoDArT marker 25,947,631) to 0.780 (for SilicoDArT marker 4,769,346), with an average of −0.310. Percentage variance accounted for particular markers varied from 6.1% (for SilicoDArT marker 4,591,564) to 7.6% (for SilicoDArT marker 4,580,898), with an average of 6.64%.

Selected markers, significant at 0.001 level with seed germination, are presented in Table 4. Effects of particular markers varied from −0.639 (for SilicoDArT marker 2,382,757) to 0.637 (for SNP marker 2,386,217), with an average of −0.143. Percentage variance accounted for particular markers varied from 6.1% (for six markers) to 10.2% (for SilicoDArT marker 2,382,757), with an average of 7.21%.

### 2.3. Physical Mapping of Gene Sequences

Fifty-six markers (forty-seven SilicoDArT and nine SNP) were significant for both traits. Of these 56 markers, the 20 most significant were selected (five of these markers were significant at the level of 0.001 for seed vigor and at the level of 0.05 for seed germination, another five markers were significant at the level of 0.001 for seed germination and at the level of 0.05 for seed vigor, five markers were significant at the level of 0.001 only for seed vigor, and five were significant at the level of 0.001 only for seed germination also selected). These markers were used for physical mapping to determine their location on the genetic map (Table 5). Six of these markers (five silicoDArT—2,435,784, 4,772,587, 4,776,334, 2,507,310, 25,981,291 and one SNP—2,386,217) are located inside genes, the action of which may affect both seed germination capacity and seed vigor (Table 5). In order to identify 20 markers significantly related to the analyzed features, primers were designed, which are presented in Table 6.

## 3. Discussion

The development of modern molecular technologies, increasing the efficiency of seed germination and seed vigor, and maximizing profits are now fundamental to sustainable agriculture. The seeds vigor and the seeds germination of maize are very complex properties of varieties genetically and physiologically determined. These features are not very easy to define, but good vigor is a feature of varieties that have more intensive initial growth and are less sensitive to frosts and also have a greater ability to uptake ingredients in low conditions temperatures. It is expressed in a greener color and less yellowing or drying of the leaves [19].

With NGS technology, we can generate huge amounts of DNA sequence data, which will become a tool for identifying high-density molecular markers. Next-generation sequencing (NGS), quantitative trait locus (QTL) mapping, genome-wide association studies (GWAS), and nested association mapping (NAM) are being used to study complex traits such as seed vigor, seed germination, yield, pathogen resistance, and control of metabolic pathways [20,21,22].

In our research, as a result of next-generation sequencing (NGS) of 152 maize lines, a total of 81,602 molecular markers (53,031 SilicoDArT and 28,571 SNP) were obtained. In the next stage of the research, these markers were used for association mapping.

For associative mapping, unrelated and differentiated in terms of the studied characteristics of the cultivar and homozygous lines are used, which are subjected to detailed phenotyping and precise genotyping. DNA profiling uses methods that scan a large part of the genome, e.g., GBS [23], DArTseq or DArT [24,25,26].

In the studies described above, 81,602 molecular markers (SilicoDArT and SNP) were used for the association mapping. Observations of phenotypic traits regarding seed vigor and seed germination capacity were also used. As a result of the association analysis, 15,409 (1559 SilicoDarT and 13,850 SNP) molecular markers significantly related to the two analyzed features were selected. The 890 molecular markers (758 SilicoDArT and 132 SNP) were associated with seed vigor and the 1323 (1115 SilicoDArT and 208 SNP) with seed germination.

Tomkowiak et al. [27], in their research, identified single-nucleotide polymorphisms (SNPs) and SilicoDarT markers related to selected morphological features affecting the yield of maize grain. Two markers were selected: marker 4,578,734 near the gene encoding anthocyanidin 3-O-glucosyltransferase and marker 4,778,900 near the gene encoding starch synthase I.

Sobiech et al. [28] identified two important markers of SiliciDArT and SNP that can be used to select fusarium-resistant cultivars. As a result of the analysis, it was found that two of the seven selected markers (15,097—SilicoDarT and 58,771—SNP) are inside genes. These markers are located on chromosomes 2 and 3, respectively. Marker 097 is anchored to the gene encoding putrescine N-hydroxycinnamyltransferase, while marker 58771 is anchored to the gene encoding the peroxidase precursor 72.

Tomkowiak et al. [29] identified new markers (SNP and SilicoDArT) for selecting maize genotypes characterized by high yields. As a result of the conducted research, six markers (1818; 14,506; 2317; 3233; 11,657; 12,812) were identified inside genes. These markers are located on chromosomes 8, 9, 7, 3, 5, and 1, respectively.

The search for molecular markers related to improvement in seed vigor under adverse condition is an important object in maize breeding currently. Higher sowing quality of seeds is necessary for the development of the agriculture production and better ability to resist all kinds of adversity in the seeds’ storage [30]. Zhang et al. [31] identified 25 QTLs associated with maize tolerance to deep seeding. The authors described 179 SSR molecular markers.

Mesocotyl length is an important feature influencing the emergence of corn seedlings after deep sowing and is closely related to abiotic stress. Zhao et al. [32] identified genes (DEGs) using RNA sequencing. They chose 15 candidate genes in the cQMES4 region that are involved in cell wall structure, lignin biosynthesis, and phytohormone signal transduction. These 15 genes can be used to study the genetic control of corn seed germination and seedling growth under deep sowing conditions.

According to Li et al. [33], oligosaccharides from the raffinose family (RFO), which accumulate in seeds during the maturation of many plant species, may be responsible for the vigor of seeds in maize.

In the research presented in this study fifty-six markers (47 SilicoDArT and nine SNP) were significant for both traits (seeds vigor and seeds germination). Of these 56 markers, the most significant 20 were selected (five of these markers were significant at the level of 0.001 for seed vigor and at the level of 0.05 for seed germination, another five markers were significant at the level of 0.001 for seed germination and at the level of 0.05 for seed vigor, five markers were significant at the level of 0.001 only for seed vigor, and five were significant at the level of 0.001 only for seed germination also selected). These markers were used for physical mapping to determine their location on the genetic map. Finally, it was found that the six of these markers (five SilicoDArT—2,435,784, 4,772,587, 4,776,334, 2,507,310, 25,981,291, and one SNP—2,386,217) are located inside genes, the action of which may affect both seed germination and seed vigor.

The SilicoDArT 2,435,784 marker is located on chromosome 1 inside the sucrose synthase 4 isoform ×2 gene. This gene is likely to have an impact on seed vigor because Stein et al. [34] showed that plants with reduced sucrose synthase (SUS) activity have reduced growth, reduced starch, cellulose or callose synthesis, reduced tolerance to anaerobic-stress conditions, and altered shoot apical meristem function and leaf morphology. Plants overexpressing SUS have shown increased growth, increased xylem area, and xylem cell-wall width, and increased cellulose and starch contents, making SUS high-potential candidate genes for the improvement of agricultural traits in crop plants. According to Zhu et al. [35], sucrose synthase is widely considered as the key enzyme involved in the plant sugar metabolism that is critical to plant growth and development, especially quality.

The molecular marker SilicoDArT 4,776,334 is located on chromosome 2 inside the phosphoinositide phosphatase sac7 isoform ×1 gene and can also affect both vigor and seed germination in maize. Research by Mao et al. [36] confirmed that the family of phosphoinositide phosphatases recently emerged as important regulators in multiple growth and developmental processes in plants. In *Arabidopsis thaliana*, two protein phosphatase type 2C (PP2C) proteins, POLTERGEIST (POL) and PLL1, regulate the asymmetric character of stem cell divisions at both the shoot and root meristems [37,38,39,40].

The molecular marker SilicoDArT 4,772,587 is located on chromosome 4 inside the putative SET domain containing protein family isoform ×1 gene. According to Thorstensen et al. [41], the majority of the histone lysine methyltransferases (HKMTases) are proteins with a conserved SET domain responsible for the enzymatic activity. The SET domain proteins in the model plant *Arabidopsis thaliana* can be assigned to evolutionarily conserved classes with different specificities allowing for different outcomes on chromatin structure. SET domain proteins are involved in developmental processes. According to Ng et al. [42] the SET domain is now recognized as generally having methyltransferase activity targeted to specific lysine residues of histone H3 or H4. There is considerable sequence conservation within the SET domain and within its flanking regions. Previous reviews have shown that SET proteins from Arabidopsis and maize fall into five classes according to their sequence and domain architectures.

The molecular marker SilicoDArT 2,507,310 is located on chromosome 2 inside the grx_c8–glutaredoxin subgroup iii gene. Glutaredoxins (GRXs) are small ubiquitous glutathione (GSH)-dependent oxidoreductases that catalyze the reversible reduction of protein disulfide bridges or protein-GSH mixed disulfide bonds via a dithiol or monothiol mechanism, respectively. A growing body of evidence indicates that plant GRXs are involved in numerous cellular pathways, including the control of flowering time and the development of postembryonic roots and shoots [43]. Glutaredoxins (Grxs) regulate several cellular processes by controlling the redox state of their target proteins. Grxs belong to thioredoxin superfamily and possess characteristic Grx fold. Several phylogenetic, biochemical, and structural studies have contributed to our overall understanding of Grxs [44].

The molecular marker SilicoDArT 25,981,291 is located on chromosome 1 inside the a-agglutinin anchorage subunit-like gene.

The molecular marker SNP 2,386,217 is located on chromosome 3 inside the probable 3-beta-hydroxysteroid-delta(8),delta(7)-isomerase gene. According to Coppola et al. [45], this gene may indirectly participate in the immune response to insects in tomatoes.

The analysis of the literature reports shows that the markers SilicoDArT 2,435,784, 4,776,334, 4,772,587, and 2,507,310 are linked to genes that can significantly affect both seed vigor and seed germination. In the next step, the expression of these genes will be analyzed in order to use them for the selection of genotypes with high seed vigor and good seed germination.

## 4. Materials and Methods

### 4.1. Plant Material

Plant material consisted of 152 inbred lines derived from Plant Breeding Smolice Sp. z o. o. IHAR Group. These lines (152) were deployed in Smolice (51°41′23.16″ N 17°4′ 18.241″ E). Inbred lines that are included in the research represent European germplasm. The first 150 inbred lines are dents (mixture of Idt and SSS heterotic groups). The last 2 inbred lines represent European flints. These two lines are used as testers for 150 dents to produce experimental hybrids.

### 4.2. Methods

#### 4.2.1. Phenotyping

The seeds’ germination was assessed in accordance with the rules of the International Seed Testing Association (ISTA) in the laboratory under optimal conditions of temperature, humidity, and quality of the germination medium (tissue paper). Under such conditions, after 14 days, the viability of the seeds was assessed because all seeds (also weak seeds) capable of germinating should germinate. The following evaluations were carried out: the first count-germination energy after 5 days, the last count-germination capacity after 10 days and vigor tests. The seedling growth test was carried out by placing 25 seeds in a roll of medium filter paper in four replications. The tissue sheets were moistened with water and placed in a thermostat at 20 °C. After completion of germination, the length of normally germinated seedlings was measured (cm) and the average length of seedlings per roll was determined. After completion of the seedling growth test, a seedling growth rate test was performed in which the normal seedlings from each roll were dried for 24 h at 80 °C, and then the weight of a single seedling was determined.

The vigor of seeds was assessed in the laboratory and directly in the field, at a relatively low temperature. The size of emergence, their speed, and homogeneity were assessed. A field experiment with 152 inbred lines was set up in 2021–2022 on plots of 10 m^2^, in a system of complete randomly selected blocks, for three repetitions.

#### 4.2.2. DNA Isolation

DNA isolation from 152 inbred lines was made by using Syngen Plant DNA MAXI Kit. (Wrocław, Poland) The concentration and purity of the isolated DNA samples were determined using a DS-11 spectrophotometer from the DeNovix company (Wilmington, NC, USA). The isolated template DNA was adjusted to an equal concentration of 100 ng/μL by dilution with distilled water.

#### 4.2.3. Genotyping

Genotyping using DArTseq technology was performed by Diversity Arrays Technology Pty Ltd. (Kirian A., Canberra, Australia). The research results were provided to the authors along with the methodology below. Analyses using the same methodology were conducted by the authors earlier and described in the publication Tomkowiak et al. [27]. The methodology in both publications is identical because it was provided to the authors each time in this form.

DNA sample digestion/ligation reactions were processed according to Kilian et al. [26], but replacing a single PstI-compatible adaptor with two adaptors corresponding to PstI- and NspI-compatible sequences and moving the assay on the sequencing platform as described by Sansaloni et al. [25]. The PstI-compatible adapter was designed to include Illumina flowcell attachment sequence, sequencing primer sequence, and “staggered” varying length barcode region, similar to the sequence reported by Elshire et al. [24]. Reverse adapter contained flowcell attachment region and NspI-compatible overhang sequence. Only “mixed fragments” (PstI–NspI) were amplified in PCR using the following reaction conditions: denaturation, 1 min at 94 °C; followed by 30 cycles of 94 °C for 20 s, 57 °C for 30 s, and 72 °C for 45 s; and the final elongation, 72 °C for 7 min. After PCR, equimolar amounts of amplification products from each sample of the 96-well microtiter plate were bulked and applied to c-Bot (Illumina) bridge PCR, followed by sequencing on Illumina Hiseq2500. The sequencing (single read) was run for 78 cycles. Sequences generated from each lane were processed using proprietary DArT analytical pipelines. In the primary pipeline, the fastq files were first processed to filter away poor-quality sequences, applying more stringent selection criteria to the barcode region compared to the rest of the sequence. In that way, the assignments of the sequences to specific samples carried in the “barcode split” step were very reliable. Approximately 2,500,000 (±7%) sequences per barcode/sample were used in marker calling. Finally, identical sequences were collapsed into “fastqcall files”. These files were used in the secondary pipeline for DArT PL’s proprietary SNP and SilicoDArT (presence/absence of restriction fragments in representation) calling algorithms (DArTsoft14). For the association analysis, only DArT sequences meeting the following criteria were selected: one SilicoDArT and SNP within a given sequence (69 nt), minor allele frequency (MAF) > 0.25, and missing observation fractions < 10%.

#### 4.2.4. Statistical Analysis and Association Mapping using GWAS Analysis

The normality of the distribution of the observed traits was tested using Shapiro–Wilk’s normality test to check whether the analysis of variance (ANOVA) met the assumption that the ANOVA model residuals followed a normal distribution. The homogeneity of variance was tested using Bartlett’s test [46]. A one-way analysis of variance (ANOVA) was carried out to determine the main effect of line on the variability of both studied traits. The relationships between seed germination and seed vigor were assessed based on Pearson’s correlation coefficients and tested with the *t*-test.

By means of GWAS analysis, an association mapping was made for the seed vigor and seed germination of 152 maize lines. This mapping was performed on the basis of the results obtained from genotyping and phenotyping. The genotypic data were obtained from the DArTseq analysis. Based on the GWAS analysis, silicoDArT and SNP markers with the highest significance level were selected for further research, that is, those that were most strongly associated with the yield structure and yield. All analyses were conducted in Genstat 18.2 (VSN International Ltd., Hemel Hempstead, UK).

#### 4.2.5. Functional Analysis of Gene Sequences

Functional analysis was performed using the Blast2GO (NCBI). The sequences of all genes located in the region of chromosomes determined by BLAST analysis conducted on the NCBI website were analyzed. The goal was to obtain information on the biological function of gene sequences located in the designated chromosome region. To determine the primers of the primers, the primer BLAST (NCBI)was used.

## 5. Conclusions

According to the assumed hypothesis in the above studies, it was possible to identify six highly important markers located inside genes. Literature reports indicate that four of these genes—namely, sucrose synthase 4 isoform ×2 gene, phosphoinositide phosphatase sac7 isoform ×1 gene, putative SET domain containing protein family isoform ×1 gene, and grx_c8–glutaredoxin subgroup iii gene—can significantly regulate the level of seed vigor and seed germination in maize. According to the assumption contained in the publication, these markers, after testing on extreme populations and fine-tuning the PCR conditions will be able to be used to select maize genotypes with higher yield potential and to determine the viability of seeds collected in Gene Banks. The results of these studies are very important from the breeding point of view, because in recent years a change in the approach to selection has been observed. Instead of using single feature markers, multiple markers of these features are searched for, or the entire pool of markers is available to describe a given population for selection purposes.

## Figures and Tables

**Table 1:** Molecular markers of SilicoDArT and SNP significantly (LOD > 3.0) related to the seed vigor and seed germination (significant associations selected at *p* < 0.05 with correction for multiple testing using the Benjamini–Hochberg method). Table contains the number of significant markers associated with each particular trait, range of significant effects (minimal and maximal effects), mean value of all significant effects, and percentage variance accounted by significant markers (minimal, maximal, and mean values).

Trait	Type of Markers		SilicoDArT	SNP
Seed vigor	The number of significant markers		758	132
	Effect	min	−0.801	−0.795
		max	0.78	0.714
		mean	0.0015	−0.036
		sum	1.106	−4.794
	Percentage variance accounted	min	2	2
		max	7.6	6.6
		mean	3.01	3.06
Seed germination	The number of significant markers		1115	208
	Effect	min	−0.639	−0.626
		max	0.621	0.637
		mean	−0.088	−0.046
		sum	−98.092	−9.591
	Percentage variance accounted	min	1.9	1.9
		max	10.2	10
		mean	3.14	3.3

**Table 2:** Molecular markers of SilicoDArT and SNP significantly (LOD > 3.0) related to both observed traits: seed vigor and seed germination (significant associations selected at *p* < 0.05 with correction for multiple testing using the Benjamini–Hochberg method).

Type of Marker	Marker	Seed Vigor		Seed Germination	
		Estimate	Percentage Variance Accounted	Estimate	Percentage Variance Accounted
SNP	2,383,357	−0.423	2.0	−0.37	3.0
DArT	2,384,346	0.431	2.1	0.332	2.3
DArT	2,394,856	−0.479	2.4	−0.337	2.1
DArT	2,427,730	−0.477	2.7	−0.316	2.0
DArT	2,435,414	0.485	2.3	0.379	2.6
DArT	2,435,784	−0.677	5.5	−0.373	2.7
DArT	2,459,805	−0.478	2.3	−0.341	2.0
DArT	2,480,018	0.459	2.2	0.331	2.0
DArT	2,496,196	−0.466	2.6	−0.371	3.0
DArT	2,500,039	−0.462	2.5	−0.42	4.0
DArT	2,512,711	0.542	2.8	0.357	2.0
DArT	2,517,355	0.525	2.9	0.402	3.1
DArT	2,523,102	−0.592	4.3	−0.36	2.6
DArT	2,524,206	0.618	4.4	0.373	2.7
DArT	2,526,601	−0.536	3.4	−0.388	3.2
DArT	2,558,379	−0.471	2.6	−0.355	2.7
SNP	2,565,188	0.487	2.2	0.378	2.5
DArT	4,576,613	0.663	5.1	0.357	2.3
DArT	4,578,890	0.695	4.8	0.386	2.4
DArT	4,584,107	−0.533	3.6	−0.373	3.1
DArT	4,584,433	−0.483	2.7	−0.318	1.9
SNP	4,584,461	0.566	3.8	0.398	3.3
DArT	4,589,607	−0.737	6.6	−0.343	2.2
DArT	4,592,681	−0.449	2.2	−0.336	2.3
DArT	4,765,935	0.441	2.1	−0.54	6.7
DArT	4,768,602	−0.44	2.0	−0.325	1.9
DArT	4,772,587	0.509	2.9	0.455	4.5
SNP	4,772,814	−0.427	2.0	−0.318	1.9
DArT	4,772,944	−0.46	2.3	−0.371	2.8
SNP	4,773,324	−0.607	4.8	−0.362	2.8
SNP	4,773,863	0.663	5.1	0.357	2.3
DArT	4,774,632	−0.518	2.7	−0.342	2.0
DArT	4,775,276	−0.455	2.4	−0.353	2.7
DArT	4,776,334	−0.651	5.2	−0.348	2.4
DArT	4,777,053	0.562	4.0	0.326	2.2
SNP	4,777,566	0.441	2.2	0.323	2.1
DArT	5585,640	−0.587	3.7	−0.34	2.0
DArT	7,049,301	−0.46	2.3	−0.343	2.3
DArT	7,050,274	−0.621	3.4	−0.4	2.4
DArT	7,054,752	−0.524	3.4	−0.329	2.2
DArT	7,059,320	0.748	6.6	0.395	3.0
DArT	7,061,686	0.493	2.9	0.32	2.0
DArT	9,625,011	−0.517	3.0	−0.343	2.3
SNP	9,687,038	−0.532	2.8	−0.364	2.3
DArT	9,693,261	−0.48	2.6	−0.36	2.6
DArT	9,710,915	0.596	4.5	0.332	2.2
DArT	16,723,050	0.498	2.8	0.369	2.7
DArT	16,725,652	0.508	2.6	0.419	3.3
DArT	16,726,826	−0.431	2.1	−0.413	3.9
DArT	21,695,758	0.459	2.2	0.331	2.0
SNP	21,698,325	−0.497	2.0	−0.383	2.1
DArT	25,000,251	−0.626	5.2	−0.371	3.0
DArT	25,943,049	0.596	3.7	0.428	3.4
DArT	25,947,240	0.548	3.1	−0.342	2.0
DArT	26,083,456	0.485	2.3	0.379	2.6
DArT	58,293,040	−0.562	4.0	−0.379	3.2

**Table 3:** Selected molecular markers (significant at the 0.001 level) related to the seed vigor.

Marker	Estimate	Percentage Variance Accounted	Marker	Estimate	Percentage Variance Accounted
4,580,898	−0.745	7.6	25,000,779	−0.762	6.6
2,507,310	0.744	7.3	9,624,535	−0.701	6.5
9,699,056	−0.719	7.0	9,687,233	0.692	6.4
25,981,291	−0.751	7.0	4,773,254	−0.788	6.4
25,947,631	−0.801	6.9	2,530,239	0.701	6.3
4,589,607	−0.737	6.6	4,582,430	−0.736	6.2
4,769,346	0.780	6.6	4,770,911	−0.736	6.2
4,779,691	−0.780	6.6	4,591,564	−0.676	6.1
7,059,320	0.748	6.6			

**Table 4:** Selected molecular markers (significant at the 0.001 level) related to the seed germination.

Marker	Estimate	Percentage Variance Accounted	Marker	Estimate	Percentage Variance Accounted
2,382,757	−0.639	10.2	2,572,299	−0.595	6.6
2,386,217	0.637	10.0	4,576,534	−0.583	6.6
4,770,719	−0.626	9.7	9,682,555	−0.521	6.6
7,059,241	0.621	9.4	24,017,322	−0.578	6.6
2,565,888	0.620	9.1	2,452,719	0.557	6.5
4,579,017	0.610	9.1	4,775,059	0.543	6.5
4,778,926	0.613	8.9	5,589,519	−0.569	6.5
4,768,869	0.599	8.8	9,679,345	−0.520	6.5
9,619,890	−0.581	8.4	16,723,671	−0.552	6.5
2,516,290	−0.599	8.3	2,387,415	0.516	6.4
4,765,379	−0.611	8.3	2,438,696	−0.535	6.4
4,779,164	0.592	8.2	4,592,741	0.548	6.4
4,582,993	0.585	8.1	2,542,705	−0.510	6.3
4,767,822	−0.577	8.1	4,584,706	−0.513	6.3
4,773,702	0.594	8.0	4,770,215	−0.514	6.3
48,474,231	−0.579	7.9	9,694,232	−0.523	6.3
4,775,452	−0.572	7.8	2,439,578	−0.528	6.2
4,775,909	−0.016	7.7	2,441,184	−0.504	6.2
4,587,134	0.571	7.5	2,509,715	−0.528	6.2
4,774,360	−0.570	7.5	4,576,581	0.517	6.2
5,584,378	−0.560	7.5	5,587,268	0.570	6.2
4,591,069	−0.572	7.4	29,622,219	−0.504	6.2
34,662,940	−0.548	7.4	2,432,805	0.521	6.1
4,777,912	−0.539	6.8	2,451,544	0.546	6.1
2,530,805	−0.542	6.7	2,610,978	0.546	6.1
4,765,935	−0.540	6.7	4,588,360	−0.504	6.1
5,589,694	−0.608	6.7	4,775,503	−0.546	6.1
2,552,487	−0.544	6.6	25,000,666	0.521	6.1

**Table 5:** Characteristics and location of markers significantly related to the analyzed traits.

Marker	Marker Type	Feature (Significant Level)	Chromosome	Marker Location	Candidate Genes
4,589,607	DArT	Seed vigor (0.001), Seed germination (0.05)	Chr2	168,930,481	128 bp at 5′ side: uncharacterized protein loc100272672 84,601 bp at 3′ side: diacylglycerol lipase-beta
7,059,320	DArT	Seed vigor (0.001), Seed germination (0.05)	Chr2	168,930,481	128 bp at 5′ side: uncharacterized protein loc100272672 84,601 bp at 3′ side: diacylglycerol lipase-beta
2,435,784	DArT	Seed vigor (0.001), Seed germination (0.05)	Ch1	56,888,568	sucrose synthase 4 isoform ×2 sucrose synthase 4 isoform ×1
4,776,334	DArT	Seed vigor (0.001), Seed germination (0.05)	Chr2	129,929,222	phosphoinositide phosphatase sac7 isoform ×1 phosphoinositide phosphatase sac7 isoform ×2
25,000,251	DArT	Seed vigor (0.001), Seed germination (0.05)	Chr1	89,047,678	110,109 bp at 5′ side: ndr1/hin1-like protein 10 552 bp at 3′ side: translocase of chloroplast 120, chloroplastic
4,765,935	DArT	Seed germination (0.001), Seed vigor (0.05)	Chr8	22,460,192	5781 bp at 5′ side: uncharacterized protein loc100279368 1404 bp at 3′ side: uncharacterized protein loc100280975
4,772,587	DArT	Seed germination (0.001), Seed vigor (0.05)	Chr4	141,172,070	putative set-domain containing protein family isoform ×1 putative set-domain containing protein family
2,500,039	DArT	Seed germination (0.001), Seed vigor (0.05)	Chr2	190,086,478	12,544 bp at 5′ side: atp-dependent dna helicase pif1-like 2947 bp at 3′ side: uncharacterized protein loc100217048
16,726,826	DArT	Seed germination (0.001), Seed vigor (0.05)	Chr2	190,140,760	821 bp at 5′ side: uncharacterized protein loc100283853 4387 bp at 3′ side: alpha carbonic anhydrase 4
25,943,049	DArT	Seed germination (0.001), Seed vigor (0.05)	Ch2	62,908,172	86,280 bp at 5’ side: uncharacterized protein loc100273593 isoform ×1 1726 bp at 3′ side: inhibitor of apoptosis-like protein
4,580,898	DArT	Seed vigor (0.001)	Ch1	159,125,630	30,617 bp at 5′ side: uncharacterized protein loc103644989 isoform ×1 161,136 bp at 3′ side: uncharacterized protein loc100274710 isoform ×2
2,507,310	DArT	Seed vigor (0.001)	Chr2	69,438,881	grx_c8–glutaredoxin subgroup iii
9,699,056	DArT	Seed vigor (0.001)	Chr1	159,575,935	228,802 bp at 5′ side: scarecrow-like protein 34 154,620 bp at 3′ side: uncharacterized protein loc103640060
25,981,291	DArT	Seed vigor (0.001)	Chr1	86,637,007	a-agglutinin anchorage subunit-like
25,947,631	DArT	Seed vigor (0.001)	Chr8	143,760,900	5439 bp at 5′ side: protein quirky 78,137 bp at 3′ side: uncharacterized protein loc100284991
2,382,757	DArT	Seed germination (0.001)	Chr3	32,582,513	672 bp at 5′ side: probable mitochondrial import receptor subunit tom20 21,113 bp at 3′ side: uncharacterized protein loc100276184
2,386,217	SNP	Seed germination (0.001)	Chr3	32,204,938	probable 3-beta-hydroxysteroid-delta(8),delta(7)-isomerase
4,770,719	SNP	Seed germination (0.001)	Chr3	32,126,239	uncharacterized protein loc100272990 uncharacterized protein loc100272990 isoform ×1
7,059,241	DArT	Seed germination (0.001)	Chr3	32,125,561	uncharacterized protein loc100272990 uncharacterized protein loc100272990 isoform ×1
2,565,888	DArT	Seed germination (0.001)	Chr3	33,897,068	11,124 bp at 5′ side: gdsl esterase/lipase at1g33811 6976 bp at 3′ side: uncharacterized protein loc100273387

**Table 6:** Primer sequences to identify markers significantly related to seed vigor and seed germination in maize.

Marker	Primer Sequences		Annual Temperature (°C)	Product Size (bp)
	Forward	Reverse		
4,589,607	ACGGGAGAGGAACGCTGCAG	GCCTAAACACAAGCAAGTGGGC	63	70
7,059,320	ACGGGAGAGGAACGCTGCAG	TCTGAAGAGCCATGGCAAAAGC	62	483
4,776,334	AACATTTACATCATCTGCAG	AATTGATCACAAATGTTATT	56	161
25,000,251	GAGAGTGCAGAGTGCAG	TGGGCATGCTACTGAGTTTT	54	207
4,765,935	AACAGACAACTACTGTAG	TCGAAACAAATTAGGATCAAACTCT	57	199
4,772,587	TACCTTGTGAAACTGCAG	ACCTGCTCGGGTCATCAAAT	52	149
2,500,039	GCTCTGTTTTCGTGCTGCAG	ACAAGATCTGTGGTGCCGAG	60	531
16,726,826	AGCCAAGGGTAGCTGCAG	CGTAGCAGCTGCATTCAAGAC	59	151
25,943,049	ATTAATAAGTGCTGCTGCAG	CGACCATTTTCGATAGCAGTA	54	74
4,580,898	ACGGTAGCAACGAACTGCAG	TACAGGTTGCAGGCTTCCAG	60	86
2,507,310	TGATGATCGAAGGGCTGCAG	TAAAGCTACTTGCGCCCACA	60	192
9,699,056	CCATCGCCATTTCCCTGCAG	TTACCCACCCCAGTACACCA	60	173
25,981,291	CTCTGCGCCTCCGTCTGCAG	AGCGCAAGCAACGTGAGAGA	62	197
25,947,631	TATCAATGTAACATCTGCAG	CCTGTTCTACTTCGTCACCGCG	60	185
2,382,757	TGCATTGCCTACATCTGCAG	GCGCAAGTAGCCCAAATACG	60	97
2,386,217	CGTACGGCCACATCCTGCAG	GGTACGCGGTGACGAAGTAG	60	78
4,770,719	CCGTACGGCCACATCCTGCAG	TTGCCGACGAAATACGCCCA	63	137
7,059,241	ATAGTAGGTGATTGCTGCAG	GGCCTGTTTGCGATTCATTT	57	153
2,565,888	TCCCCACAGCACAGCTGCAC	CCGGTTCAGTTTTTCCGGCG	62	88
